# In Memoriam

**Published:** 1888-12

**Authors:** 


### IN MEMORIAM.

At the regular meeting of the Brownsville and Matamoras Medical Society, held at the office of Dr. Arthur S. Wolf, on the 2nd of November, 1888, the following resolutions were passed :

WHEREAS, Death has removed from among us our friend, and colleague, Etienne Melou, M. D.

*Resolved*, That the members of the Brownsville and Matamoras Medical Society, of which he was a member and indentified from its inception, wish to express our deep sorrow, for his untimely death.

*Resolved*, That in the life and character of our departed friend, we recognize with grateful recollection, his never swerving upright and honorable conduct, both privately and professionally, and that his death will leave a void in our ranks much to be regretted.

*Resolved*, That in the death of Etienne Melou, M. D., we lament the loss of one distinguished no less for his professional attainments than for his kindness of heart.

*Resolved*, That in the discharge of his professional duties, he brought a conscientious fidelity which endeared him to his fellow-men; he will be long remembered with deep affection by rich and poor alike.

*Resolved*, That a copy of these resolutions be published in the medical journals of the state.

MIGUEL CICERO, M. D.,
ARTHUR S. WOLFF, M. D.,
J. M. MAIN, M. D.,
SAM B. ROBINSON, Asst. Surg. U. S. A.,
J. A. WOLF, Act. Asst. Surg. U. S. A.,
CHAS. MACMANUS, M. D.

Dr. Etienne Melou was born on the 23d of December, 1833, in Toulouse, France, graduated at New Orleans, 1869, at the Tulane University, Louisiana, was a member of the "State of Texas Medical Association," and of the Brownsville and Matamoras Medical Society. A. S. W.

"ONE MORE UNFORTUNATE."—Dr. Z. W. Baker, of Temple, Texas, committed suicide on the 13th instant, in a cell of the calaboose in Temple. The doctor had just returned from Fort Worth, where, it is said, he imbibed too freely of the "inspiration fountain," for which that city is famous, and returning to Temple, he drove "with a looseness" through the streets, for which he was arrested and imprisoned. To allay the effects of the whisky, with which, the telegram states, he was crazed, he injected a powerful dose of atropia and morphine into his arm, and died from the effects of it. This is a most deplorable occurrence. Dr. Baker was a much respected and prominent physician, a member of the Texas State Medical Association, and had been married to his young wife only about twelve months. It does seem to us that his many friends should have cared for him, and the authorities should have searched his person for anything with which he could have inflicted injury on himself, seeing the crazed state in which he was at the time of arrest.

---

A "REVIEW of the Austin Lunatic Asylum and its Management by Governors, Trustees and Superintendents," will be given our readers in a forthcoming number of this JOURNAL. It will be written by one fully competent to the delicate undertaking, and who, being perfectly disinterested, yet having the welfare of the unfortunates of Texas largely at heart,—will do *all parties* justice; one who is as capable of recognizing merit and appreciating fidelity to trust, as of seeing and censuring the lack of it. It will be written by a man of cool, deliberate judgment, who does nothing in a hurry, and who will "nothing extenuate, naught set down in malice." Our readers may anticipate a paper of great and thrilling interest.

---

DR. MCKENZIE, it is reported, has resigned his membership in the Royal College of Physicians and Surgeons in consequence of the adoption of a resolution by a committee of the British Medical Association, pronouncing the publication of the details of the case of Frederick a breach of professional confidence and secrecy.

